# Supplementary material for: Coordinating principal–agent and incentive strategy of cold chain logistics service in fresh food supply chain
Source: PLoS One. 2024 Oct 4;19(10):e0306976. doi: 10.1371/journal.pone.0306976 (PMC11452009; doi:10.1371/journal.pone.0306976)
Supplement: S1 Appendix — (DOCX) [file pone.0306976.s001.docx]

**Appendix**

Proof of Proposition 1: ${e_{1}^{P}}^{*}-{e_{1}^{B}}^{*}=\frac{r_{1}\sigma_{1}^{2}\gamma_{1}}{r_{1}\sigma_{1}^{2}b_{1}+{\gamma_{1}}^{2}}>0,{e_{2}^{P}}^{*}-{e_{2}^{B}}^{*}=\frac{r_{2}\sigma_{2}^{2}\gamma_{2}}{r_{2}\sigma_{2}^{2}b_{2}+{\gamma_{2}}^{2}}>0$, in summary，${e_{n}^{P}}^{*}>{e_{\begin{aligned} &n \\ & \end{aligned}}^{B}}^{*}$,end of the proof.

Proof of Proposition 2: $\frac{\partial{e_{1}^{P}}^{*}}{\partial\gamma_{1}}=\frac{1}{b_{1}}>0,\frac{\partial{e_{2}^{P}}^{*}}{\partial\gamma_{2}}=\frac{1}{b_{2}}>0,\frac{\partial{e_{1}^{B}}^{*}}{\partial\gamma_{1}}=\frac{\gamma_{1}}{b_{1}}>0,\frac{\partial{e_{2}^{B}}^{*}}{\partial\gamma_{2}}=\frac{\gamma_{2}}{b_{2}}>0,\frac{\partial e^{Pc^{*}}}{\partial\gamma}=\frac{1}{b}>0,\frac{\partial\beta^{Bc^{*}}}{\partial\gamma}=\frac{\beta^{Bc}}{b}>0,\frac{\partial e_{1}^{P*}}{\partial b_{1}}=-\frac{\gamma_{1}}{b_{1}^{2}}<0$ ,$\frac{\partial{e_{2}^{P}}^{*}}{\partial b_{2}}=-\frac{\gamma_{2}}{b_{2}^{2}}<0,\frac{\partial{e_{1}^{B}}^{*}}{\partial b_{1}}=-\frac{\beta_{1}^{B}\gamma_{1}}{b_{1}^{2}}<0，\frac{\partial{e_{2}^{B}}^{*}}{\partial b_{2}}=-\frac{\beta_{2}^{B}\gamma_{2}}{b_{2}^{2}}<0，\frac{\partial e^{Pc^{*}}}{\partial b}=-\frac{\gamma+\tau}{b^{2}}<0$, $\frac{\partial{e^{Bc}}^{*}}{\partial b}=-\frac{\beta^{Bc}\left( \gamma+\tau\right)}{b^{2}}<0$, in summary，$\frac{\partial e^{*}}{\partial\gamma}>0$, $\frac{\partial e^{*}}{\partial b}<0$，end of the proof.

Proof of Proposition 3: ${\beta_{1}^{P}}^{*}={\beta_{2}^{P}}^{*}={\beta^{Pc}}^{*}=0$, from this, we can end of the proof.

Proof of Proposition 4: $\frac{\partial\beta_{1}^{B^{*}}}{\partial\gamma_{1}}>0,\frac{\partial\beta_{2}^{B^{*}}}{\partial\gamma_{2}}>0,\frac{\partial\beta^{Bc^{*}}}{\partial\gamma}>0,\frac{\partial\beta_{1}^{B^{*}}}{\partial b_{1}}<0,\frac{\partial\beta_{2}^{B^{*}}}{\partial b_{2}}<0,\frac{\partial{\beta^{Bc}}^{*}}{\partial b}<0,\frac{\partial{\beta_{1}^{B}}^{*}}{\partial r_{1}}<0，\frac{\partial{\beta_{2}^{B}}^{*}}{\partial r_{2}}<0,\frac{\partial{\beta^{Bc}}^{*}}{\partial r}<0,$ in summary，$\frac{\partial\beta^{*}}{\partial\gamma}>0$，$\frac{\partial\beta^{*}}{\partial b}<0$，end of the proof.

Proof of Proposition 5: The process is similar to Proposition 10, which leads to $\frac{\partial Y}{\partial b}<0,\frac{\partial Y}{\partial\gamma}>0$，end of the proof.

Proof of Proposition 6:${Y_{1}^{B}}^{*}-{Y_{1}^{P}}^{*}=\frac{r_{1}{\gamma_{1}}^{2}\sigma_{1}^{2}}{2r_{1}\sigma_{1}^{2}b_{1}+2{\gamma_{1}}^{2}}>0$，${Y_{2}^{B}}^{*}-{Y_{2}^{P}}^{*}=\frac{r_{2}{\gamma_{2}}^{2}\sigma_{2}^{2}}{2r_{2}\sigma_{2}^{2}b_{2}+2{\gamma_{2}}^{2}}>0$, ${Y^{Bc}}^{*}-{Y^{Pc}}^{*}=\frac{r\left( \gamma+\tau\right)^{2}\sigma^{2}}{2r\sigma^{2}b+2\left( \gamma+\tau\right)^{2}}>0$，in summary，${Y_{1}^{B}}^{*}>{Y_{1}^{P}}^{*}$ ，${Y_{2}^{B}}^{*}>{Y_{2}^{P}}^{*}$，${Y^{Bc}}^{*}>{Y^{Pc}}^{*}$，end of the proof.

Proof of Proposition 7: we can set $\gamma=\gamma_{1}=\gamma_{2}$，$b=b_{1}=b_{2}$,which leads to ${e^{Pc}}^{*}-{e_{1}^{P}}^{*}=\frac{\gamma}{b}>0$，${e^{Pc}}^{*}-{e_{2}^{P}}^{*}=\frac{\gamma}{b}>0$，in summary，${e^{Pc}}^{*}>{e_{1}^{P}}^{*}$，${e^{Pc}}^{*}>{e_{2}^{P}}^{*}$，the proof is complete.

Proof of Proposition 8: we can set $\gamma=\gamma_{1}=\gamma_{2}$，$b=b_{1}=b_{2}$，$r=r_{1}=r_{2}$，$\sigma=\sigma_{1}=\sigma_{2}$,which leads to${\beta^{Bc}}^{*}-{\beta_{1}^{B}}^{*}={\beta^{Bc}}^{*}-{\beta_{2}^{B}}^{*}=\frac{\left( 2\gamma+\tau\right)b\sigma^{2}r\tau}{\left( r\sigma^{2}b+\left( \gamma+\tau\right)^{2} \right)\left( r\sigma^{2}b+\gamma^{2} \right)}>0,in summary，{\beta^{Bc}}^{*}>{\beta_{1}^{B}}^{*}$，${\beta^{Bc}}^{*}>{\beta_{2}^{B}}^{*}$，the proof is complete.

Proof of Proposition 9: The process is similar to Proposition 6，in summary, $\frac{\partial{e^{Pc}}^{*}}{\partial\tau}>0，\frac{\partial{e^{Bc}}^{*}}{\partial\tau}>0$，$\frac{\partial e^{*}}{\partial\tau}>0$，the proof is complete.

Proof of Proposition 10: The process is similar to Proposition 6， in summary， $\frac{\partial{\beta^{Bc}}^{*}}{\partial\tau}>0$，the proof is complete.

Proof of Proposition 11: $\frac{\partial{Y^{Pc}}^{*}}{\partial\tau}=\frac{\gamma+\tau}{b}>0,\frac{\partial{Y^{Bc}}^{*}}{\partial\tau}=\frac{\left( \gamma+\tau\right)^{3}\left( 2r\sigma^{2}b+\left( \gamma+\tau\right)^{2} \right)}{b\left( r\sigma^{2}b+\left( \gamma+\tau\right)^{2} \right)^{2}}>0$，in summary $\frac{\partial Y}{\partial\tau}>0$，the proof is complete.

Proof of Proposition 12: we can set $\gamma=\gamma_{1}=\gamma_{2}$，$b=b_{1}=b_{2}$，${Y^{Pc}}^{*}>{Y_{1}^{P}}^{*}$+${Y_{2}^{P}}^{*}$，This yields the inequality $\frac{\left( \gamma+\tau\right)^{2}}{2b}>\frac{\gamma_{1}^{2}}{2b_{1}}+\frac{\gamma_{2}^{2}}{2b_{2}}$，the range of values of τ is obtained after simplifying the inequality $\tau>\left( \sqrt{2}-1 \right)\gamma$. In the same way，we can set ${Y^{Pc}}^{*}<{Y_{1}^{P}}^{*}$+${Y_{2}^{P}}^{*}$，The range of values of τ is obtained after simplifying the inequality $0<\tau<\left( \sqrt{2}-1 \right)\gamma$，the proof is complete.
