# Supplementary material for: Coordinating principal–agent and incentive strategy of cold chain logistics service in fresh food supply chain
Source: PLoS One. 2024 Oct 4;19(10):e0306976. doi: 10.1371/journal.pone.0306976 (PMC11452009; doi:10.1371/journal.pone.0306976)
Supplement: S1 Table — (DOCX) [file pone.0306976.s002.docx]

**Table 1 Related Parameter Setting**

| Variables | Parameters | Parameter descriptions |
| --- | --- | --- |
| Exogenous variables  Exogenous variables | $b$ | Cost of effort factor for CCL service providers |
|  | $\gamma$ | Effort output factor of CCL service |
|  | $\omega$ | Retained utility of CCL service |
|  | $r$ | Absolute risk aversion factor for CCL service providers |
|  | $\theta$ | Exogenous random variable |
|  | $x$ | Actual money income |
|  | $\alpha$ | Fixed compensation |
|  | $\sigma^{2}$ | Variance of observable information |
|  | $\tau$ | Coefficient of synergies |

| Decision variables | $e$ | Level of effort of CCL service providers |
| --- | --- | --- |
|  | $\beta$ | Incentive intensity factor for fresh food producers or distributors |
| Function | $\pi$ | Expected revenue function for fresh food producers or distributors |
|  | $Y$ | Expected net revenue function for fresh food producers or Distributors |
|  | $s$ | Expected return function of CCL service providers |
|  | $Z$ | Expected net return function for CCL service providers |
|  | $C$ | Effort cost function for CCL service providers |
| Top and bottom labels | $\left( \right)_{n}^{i}$ | $i\in\left\{ P,B \right\}$ represents the full information condition versus the incomplete information condition; $c$ represents the existence of synergy between the principals; $n\in\left\{ 1,2 \right\},$where 1 is a fresh food producer and 2 is a fresh food distributor. $P$ represents the base case for full information conditions, $B$ represents the base case for incomplete information conditions, $Pc$ represents the condition of collaborative cooperation among principals under conditions of full information, $Bc$ represents the condition of collaborative cooperation among principals under conditions of incomplete information. |
