# Supplementary material for: Coordinating principal–agent and incentive strategy of cold chain logistics service in fresh food supply chain
Source: PLoS One. 2024 Oct 4;19(10):e0306976. doi: 10.1371/journal.pone.0306976 (PMC11452009; doi:10.1371/journal.pone.0306976)
Supplement: S1 Fig — (DOCX) [file pone.0306976.s003.docx]

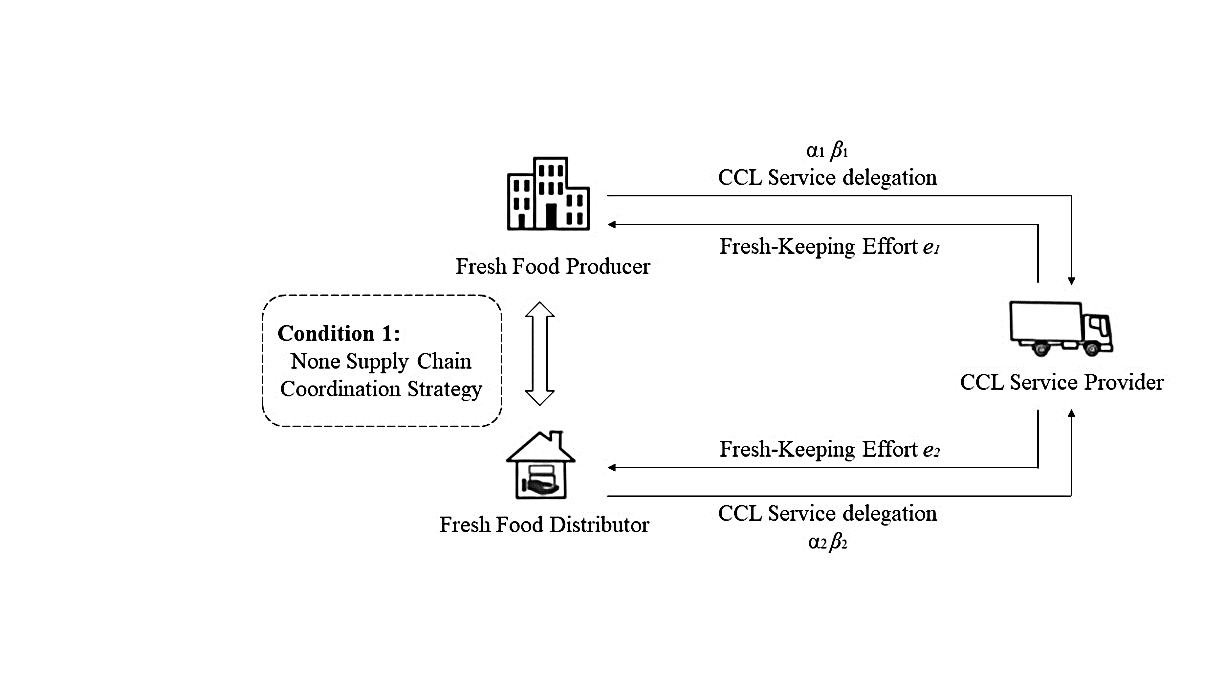


**Figure 1 Structure of the principal-agent base model of cold chain service of fresh produce supply chain**


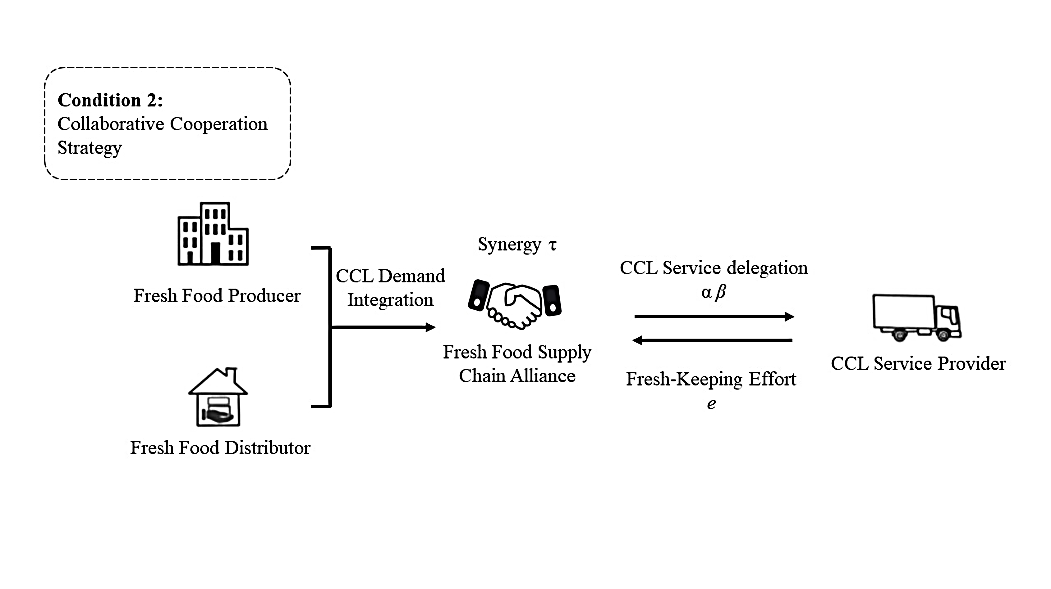


**Figure 2 Structure of the principal-agent model of cold chain service considering synergy effect**


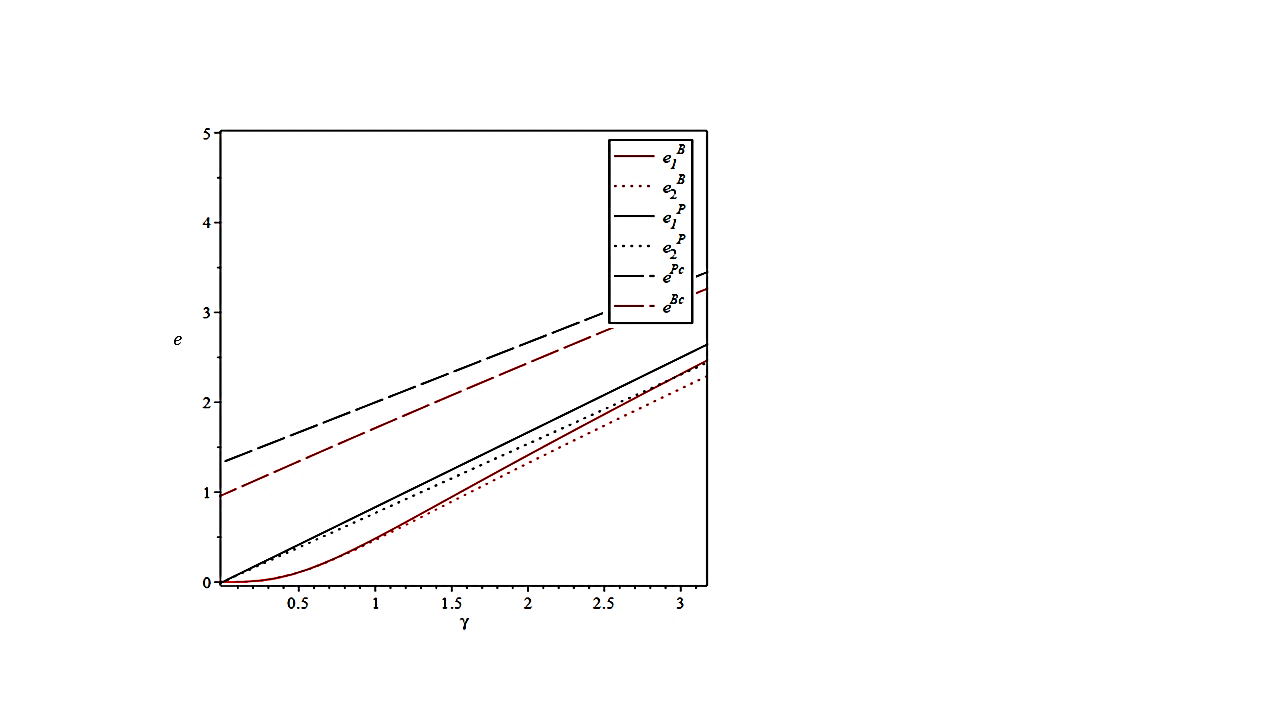


**Figure 3 Effect of Effort Output Coefficient on Level of Effort**


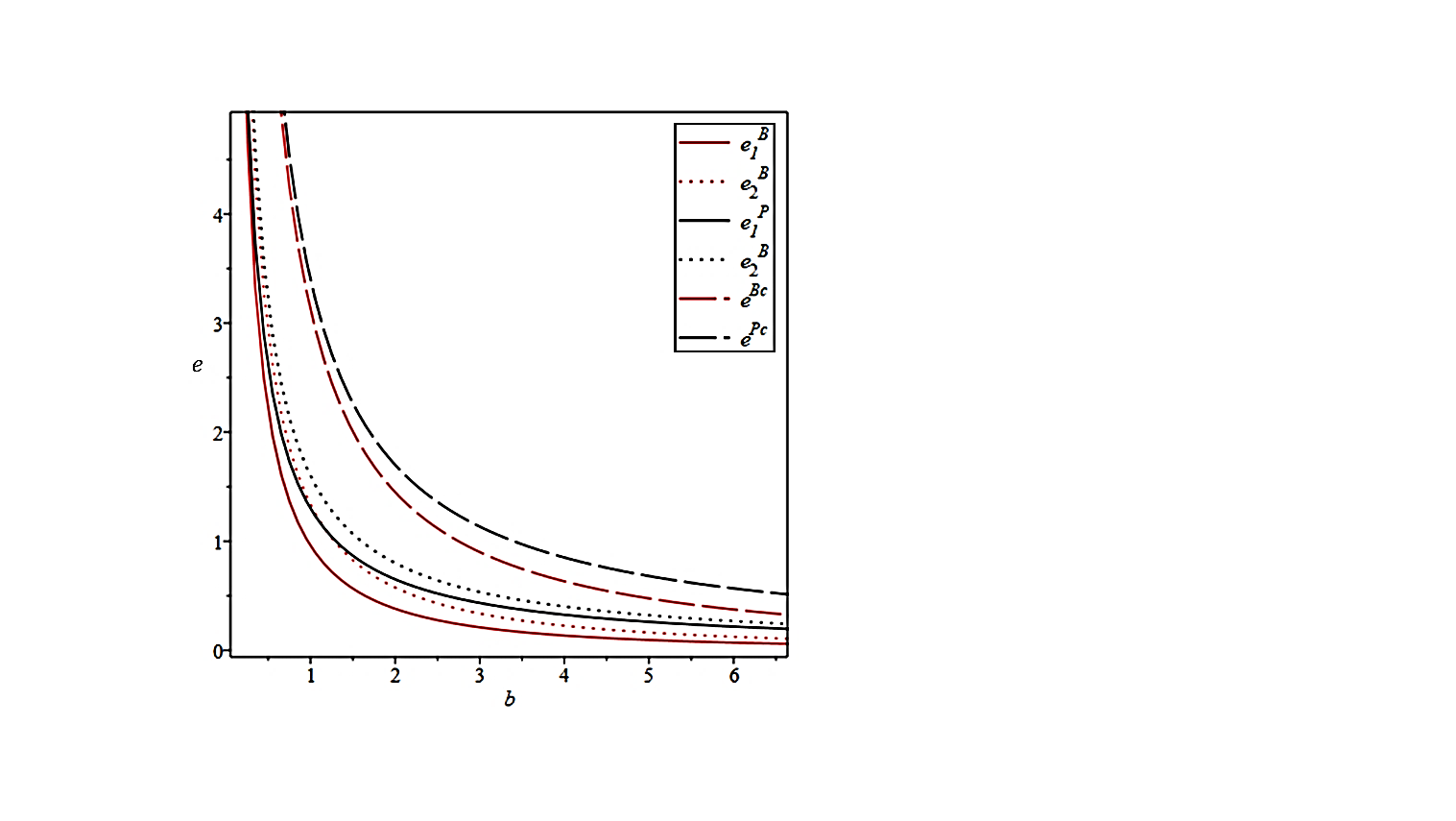


**Figure 4 Effect of Effort Cost Coefficients on Effort Levels**


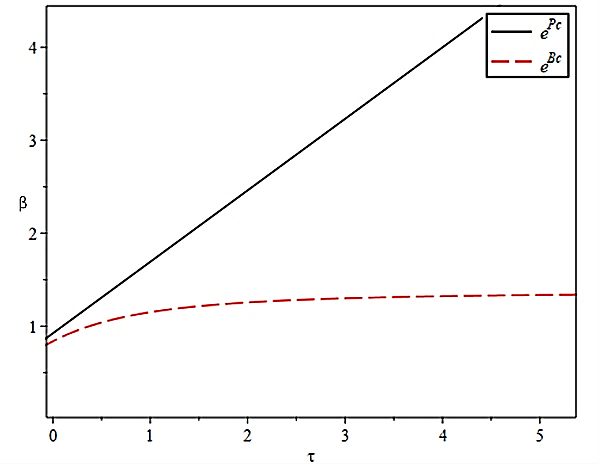


**Figure 5 Impact of synergy coefficients on the level of effort**


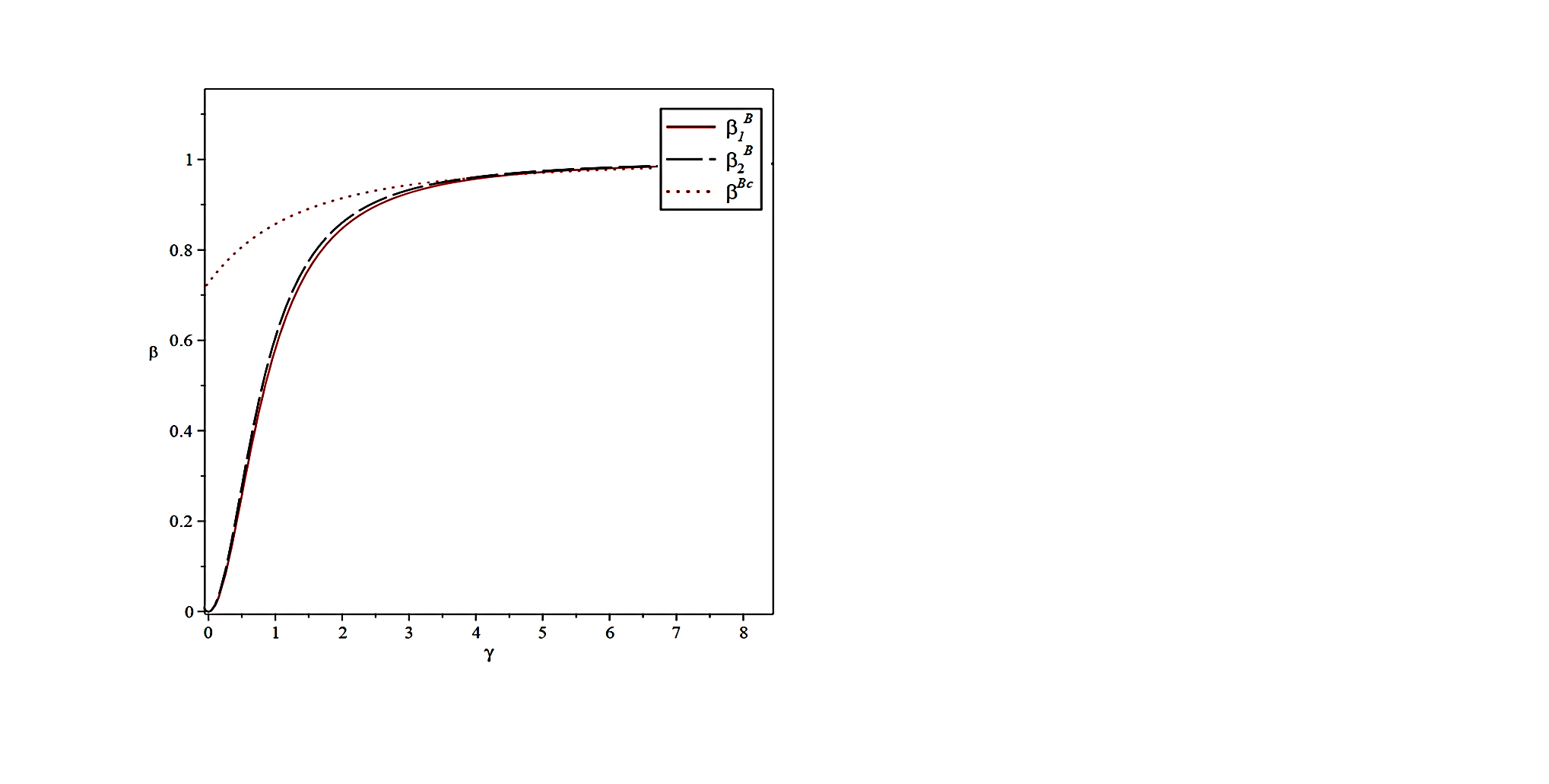


**Figure 6 Effect of Effort-Output Coefficients on Incentive Strategies**


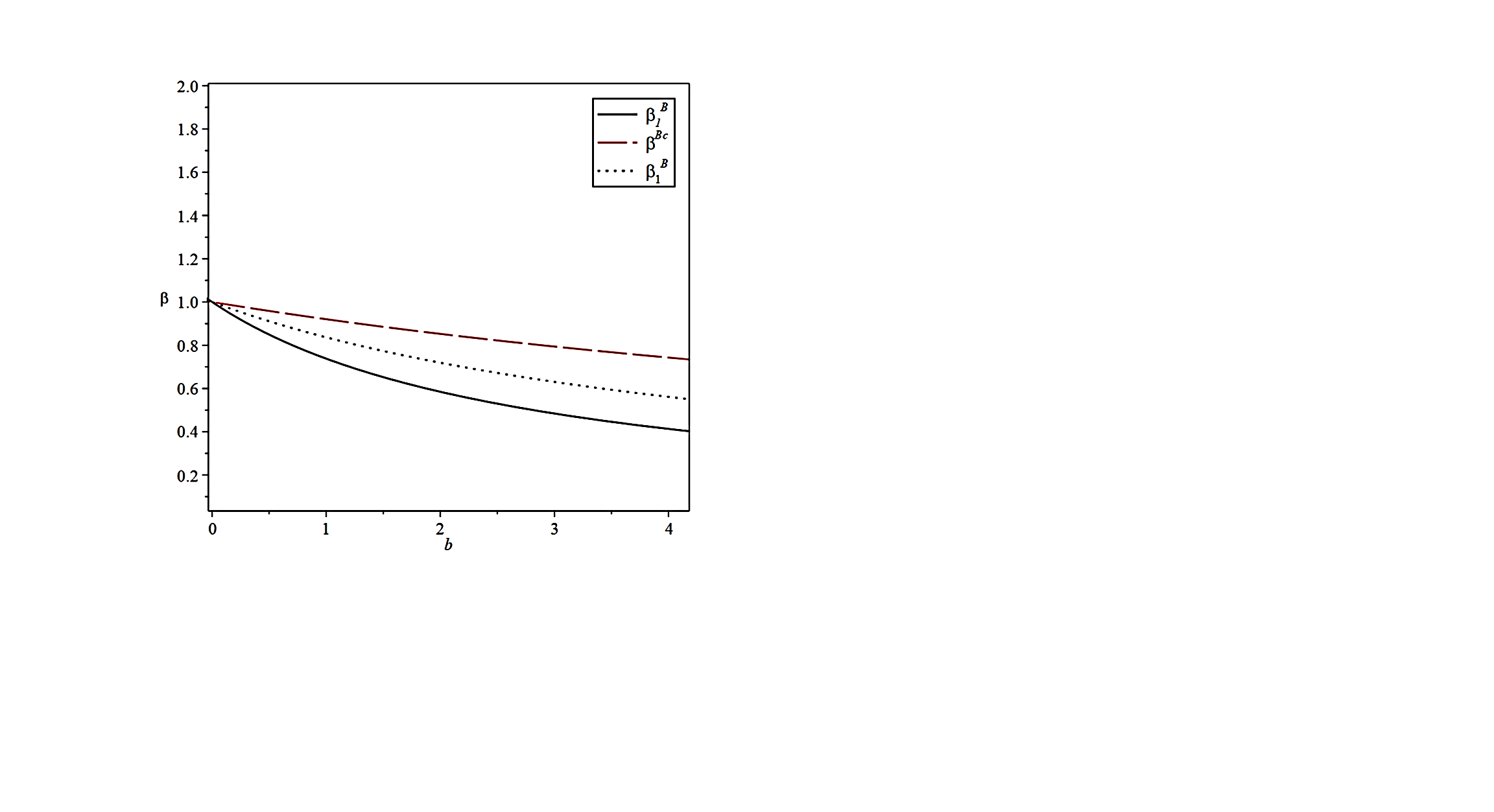


**Figure 7 Effect of Cost of Effort Coefficient on Incentive Strategies**


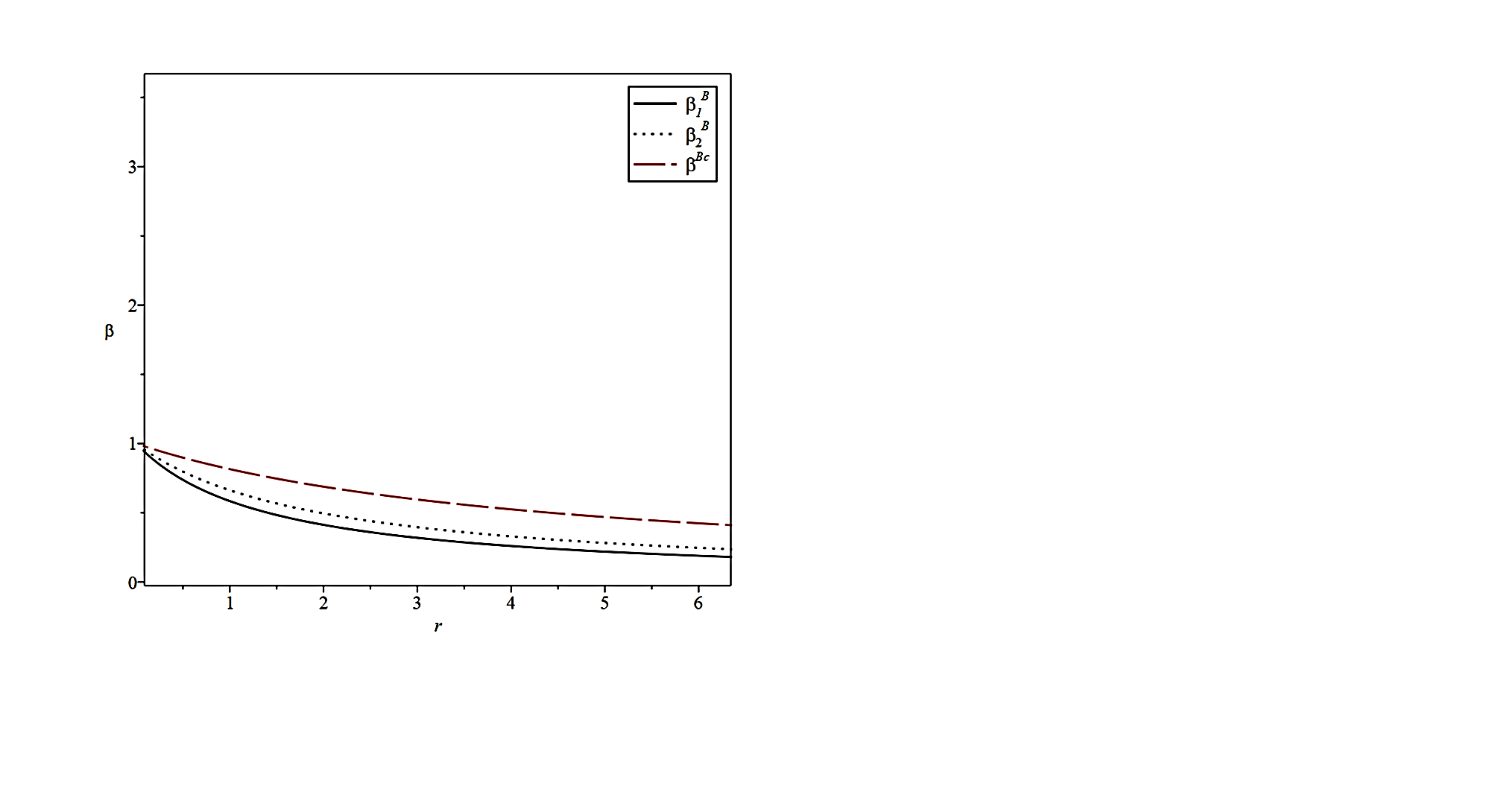


**Figure 8 Effect of Risk Aversion Coefficient on Incentive Strategies**


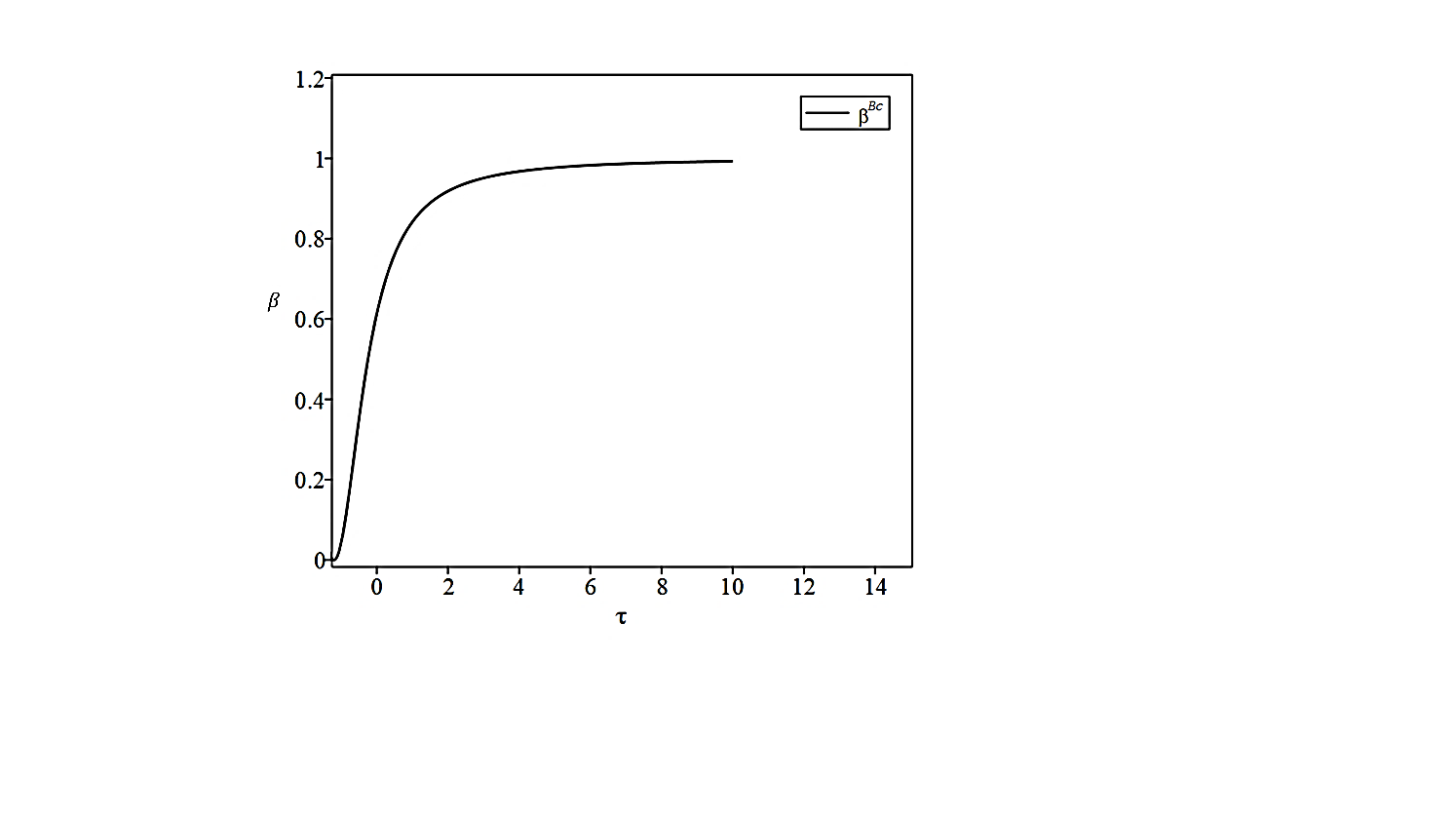


**Figure 9 Effect of Synergy Benefit Coefficients on Incentive Strategies**


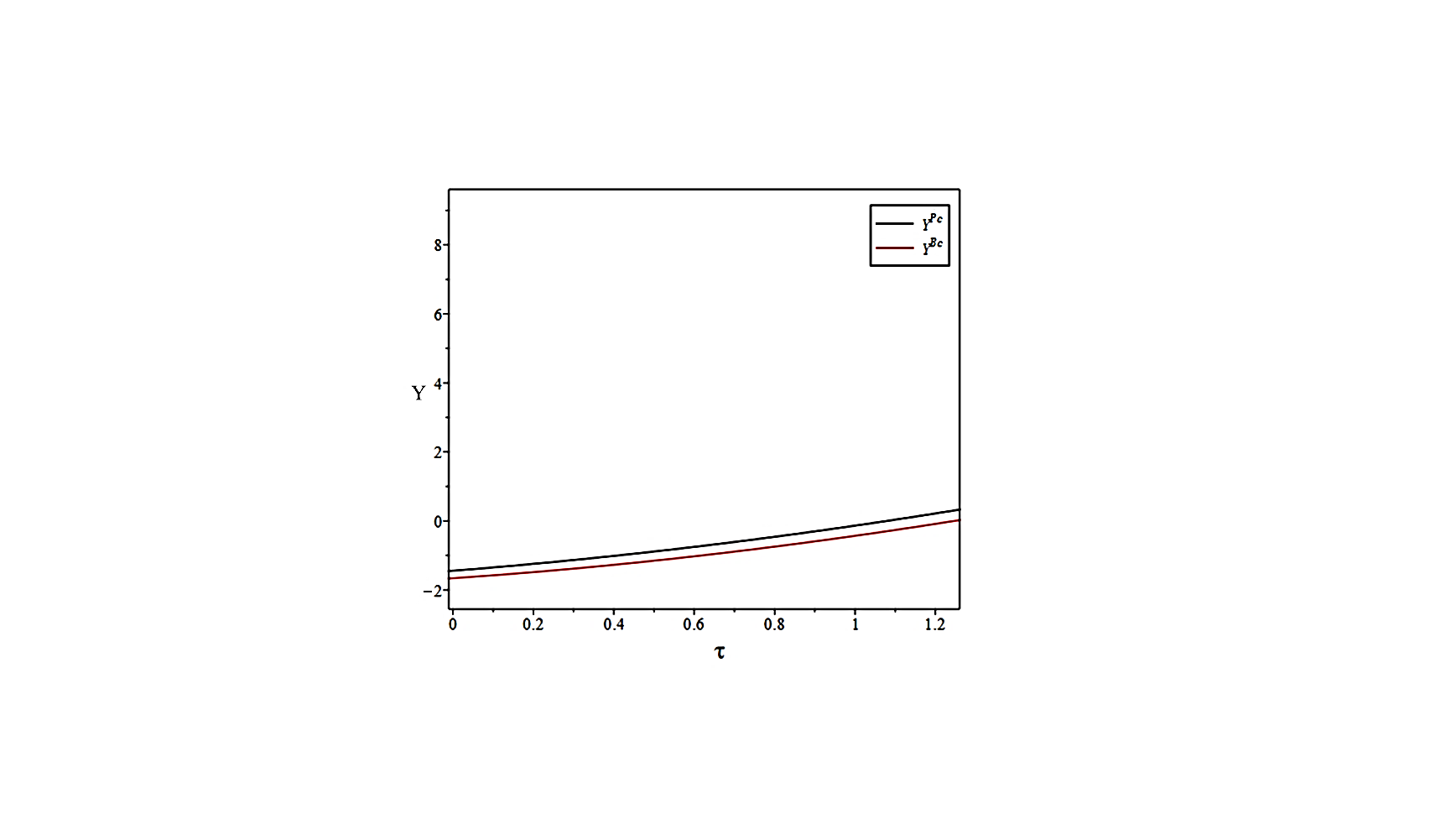


**Figure 10 Effect of synergy coefficients on preservation gains**
