# Supplementary material for: Coordinating principal–agent and incentive strategy of cold chain logistics service in fresh food supply chain
Source: PLoS One. 2024 Oct 4;19(10):e0306976. doi: 10.1371/journal.pone.0306976 (PMC11452009; doi:10.1371/journal.pone.0306976)
Supplement: Attachment — Submitted filename: Response to Reviewers.docx [file pone.0306976.s004.docx]

**Cover Letter**

Date: 15 March 2024.

Dear editor,

Thank you and the reviewers for commenting on our manuscript (PONE-D-23-32982) entitled “Coordinating principal-agent and incentive strategy of cold chain logistics service in fresh food supply chain”. We revised the manuscript in accordance with the reviewers’ comments, and highlighted the modified parts with a different color.

Reviewer 1

1. For the studied problem, are there any other existing studies via coordination theory? If yes, please include them in your literature review. If possible, it is better to compare the performance of the suggested method and theirs.

Reply:

Thanks for the reviewer’s suggestion. Some representative studies via coordination theory have been added into the literature review of our revised manuscript. Also, the performance of the suggested method and the theirs have been compared comprehensively. For further details, please refer to the first and second paragraphs of section 2.3 Supply chain coordination and incentive strategies of the literature review.

2. It is missing an in-depth discussion considering this study’s findings and how they advance the literature.

Reply:

The findings of our manuscript were explained thoroughly and an in-depth discussion about how they advance the literature were conducted. Please see the third paragraph of the final section 2.3 Supply chain coordination and incentive strategies of the literature review.

3. Please highlight the contribution of this study, with reference to practice and contribution to the literature.

Reply:

The contributions of this study have been highlighted and clearly divided into theoretical and practical parts. The details can be found from the ninth paragraph of the introduction.

4. All figures should be improved for better reading.

Reply:

All figures have been modified from the aspects of clarity, color, and line pattern for better reading. All images in our submitted revisions are attached separately.

5. English writing must be well-polished.

Reply:

Thanks for this comment. The English expression of the full manuscript has been thoroughly checked and revised, all the changes were marked in red.

Reviewer 2

1. The paper contains too many grammatical errors, spelling errors, punctuation errors, and formatting errors. Therefore, the authors are suggested to scan the entire text for possible English errors and rectify those accordingly. In addition, I think the writing of this paper could be improved in terms of logicality.

Reply:

Thanks a lot for the comments. The grammatical errors, spelling errors, punctuation errors, and formatting errors were carefully checked and the full text was examined in detail. Beyond, the logic of the writing was reorganized.

2. In Section 1, the authors are advised to provide answers to the research questions.

Reply: Thank you for the suggestion. The answers of the three research questions were provided at the end of the Introduction Section.

3. Why are Food Producer and Distributor both risk neutral while CCL Service Provider is risk averse? Please explain.

Reply: The Food Producer and Distributor both risk neutral while CCL Service Provider is risk averse is based on the second hypothesis in our manuscript, which relies on the classic HM principal-agent theory elaborated in references Bernheim&Whitnston, 1985 and Mirrlees, 1976, as well as the traditional rational person hypothesis, which assumes that the principal, i.e. fresh agricultural product producers and distributors, is risk neutral and completely rational, while the agent, i.e. cold chain logistics service provider, is completely rational and risk averse. The above literatures have been added to the revised manuscript and explained in details. Please see Assumption 2 in Section 3.1.

4. The authors actually set up the model based on the assumption in which costs except effect cost in the supply chain are standardized to 0. However, this assumption is not clearly demonstrated in Section 3, although the assumption is common in the existing literature (e.g., references [1] and [2]). Thus, the authors are advised to add the assumption and cite references [1] and [2].

Reply: The hypothesis "costs except effect cost in the supply chain are standardized to 0" has been clearly demonstrated in Section 3 and the suggested references were cited in the revised version. The details can refer to Assumption 6 in Section 3.1.

5. Please provide reasons of each proposition in detail, rather than just paraphrasing the meaning of each proposition.

Reply:

The detailed information on how each proposition was derived has been supplied in the manuscript, please see XXX.

6. It is recommended that the Conclusion section should not exceed four paragraphs.

Reply:

Thanks for the suggestion. The conclusion section has been reorganized and merge into three paragraphs. Please refer to Section 6.1.

7. It is recommended that the authors provide future research in Section 6.

Reply:

The future scope of research has been extensively discussed in Section 6.2 in our revised manuscript. .

All the contents indicated above are in the revised manuscript. In summary, we appreciate the reviewers greatly for all the comments and suggestions, which are valuable in improving the quality of our manuscript.

Yours Sincerely,

Yuxiang Wu

College of Management Science

Chengdu University of Technology, China.
